# Human myiasis caused by the reindeer warble fly, *Hypoderma tarandi,* case series from Norway, 2011 to 2016

**DOI:** 10.2807/1560-7917.ES.2017.22.29.30576

**Published:** 2017-07-20

**Authors:** Jörgen Landehag, Andreas Skogen, Kjetil Åsbakk, Boris Kan

**Affiliations:** 1 Department of Paediatrics, Finnmark Hospital Trust, Hammerfest, Norway; 2 UiT The Arctic University of Norway, Department of Arctic and Marine Biology, Tromsø, Norway; 3 Unit of Infectious Diseases, Department of Medicine, Karolinska Institutet, Stockholm, Sweden

**Keywords:** *Hypoderma tarandi*, *Rangifer tarandus* spp., human myiasis, ophthalmomyiasis, children, periorbital oedema, dermal swellings, lymphadenopathy

## Abstract

*Hypoderma tarandi* causes myiasis in reindeer and caribou (*Rangifer tarandus* spp.) in most northern hemisphere regions where these animals live. We report a series of 39 human myiasis cases caused by *H. tarandi* in Norway from 2011 to 2016. Thirty-two were residents of Finnmark, the northernmost county of Norway, one a visitor to Finnmark, and six lived in other counties of Norway where reindeer live. Clinical manifestations involved migratory dermal swellings of the face and head, enlargement of regional lymph nodes, and periorbital oedema, with or without eosinophilia. Most cases of human myiasis are seen in tropical and subtropical countries, and in tourists returning from such areas. Our findings demonstrate that myiasis caused by *H. tarandi* is more common than previously thought. Healthcare professionals in regions where there is a likelihood of human infestation with *H. tarandi* (regions populated by reindeer), or treating returning travellers, should be aware of the condition. All clinicians are advised to obtain a detailed travel history when assessing patients with migratory dermal swellings. On clinical suspicion, ivermectin should be given to prevent larval invasion of the eye (ophthalmomyiasis). Since *H. tarandi* oviposits on hair, we suggest wearing a hat as a prevention measure.

## Introduction

Myiasis is the condition where fly larvae have infested a mammal and feed on its tissues. Cutaneous myiasis can be subdivided into furuncular, migratory (creeping) and wound myiasis [1,2]. Cutaneous myiasis, the most common form in humans, is endemic in poor populations of many tropical and subtropical countries [3,4]. In Europe, myiasis is a relatively common travel-associated skin condition [1,2]. Migratory myiasis in humans is caused by *Gasterophilus* and *Hypoderma* flies (order Diptera, family Oestridae) [5]. *Hypoderma tarandi* causes myiasis in reindeer and caribou in most northern hemisphere regions (Alaska, Canada, Nordic countries in Europe including Greenland (self-governing territory of Denmark) and Russia) where these animals live [5].

Most of the 24 human cases of myiasis caused by *H. tarandi* reported in Norway and other countries in the literature from 1982 to 2016 [6-17] developed *ophthalmomyiasis interna* (OMI), a condition where the larva has invaded the eye globe, often leading to visual impairment. Most of them were visitors to northern parts of Norway and Sweden. Two cases were from Nunavut (northern Canada) [13] and one from Greenland [17]. Here we present clinical and epidemiological features from a large case series of an additional 39 consecutive human cases of myiasis caused by *H. tarandi*, mostly in children in northern Norway, from 2011 to 2016.

## Methods

### Recruitment of cases and setting

Patients with suspected myiasis (transitional dermal swellings of the face or head, history of residence in or visit to a reindeer-inhabited area in summer or autumn) were recruited from August 2011 to October 2016. Most were recruited by the Department of Paediatrics, Finnmark Hospital Trust (Hammerfest, Finnmark) and by primary care physicians in Finnmark, the remainder by primary care physicians in other counties of Norway. A blood sample was taken for serological assaying. Diagnosis was based on clinical manifestations, serology and other findings. Data regarding baseline characteristics (age, sex, occupation, residence and clinical history) were collected, and consent to publish case details was obtained from each patient. The patients were diagnosed and managed consecutively as they were recruited.

After the first three cases were diagnosed in early November 2012 (symptom onset September–November 2012), we sent a letter about *H. tarandi* myiasis in early December 2012 to inform primary care physicians in Finnmark, and the Departments of Paediatrics and Ophthalmology at the University Hospital of North Norway (UNN), Trømsø. News media, such as NRK Sápmi (a Sami-language television broadcasting service for Norway, Finland and Sweden), promoted information about the disease in December 2012. Other news media in Norway, Finland and Sweden also informed the public in 2013, and authors of this paper and others described the condition at medical meetings (Norway and Sweden) and reindeer husbandry conferences (Norway and Finland). One of the adult cases, who visited Finnmark to hunt in August 2012, was from a county in southern Norway where there are no reindeer (symptom onset September 2012, sought medical attention after receiving information via media). Similarly, two children in Finnmark, with myiasis symptom onset in September and November 2011, respectively, received information via media, and were shown to be seropositive and diagnosed more than a year after symptom onset.

### Laboratory testing

**Figure 1:**
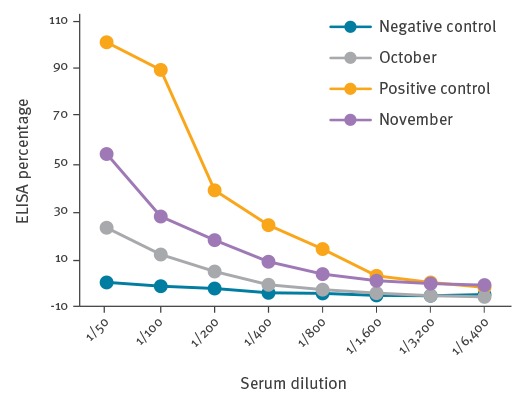
Serum samples from an 8-year-old child with myiasis caused by *Hypoderma tarandi* infestation, Norway, 2013

Serum was assayed (ELISA) for antibodies (IgG) against Hypodermin C (HyC, a collagenolytic enzyme secreted by *Hypoderma* spp. larvae during migration in the host), essentially as described for sera from reindeer [18]. Briefly, 96-well microtitre plate wells (Nunc PolySorp, Nunc, Roskilde, Denmark) were coated (0.5 µg/ml of HyC in 0.1 M sodium carbonate buffer, pH 9.6, 1 hour at 37 °C) and blocked (5% skimmed milk powder (Merck KGaA, Darmstadt, Germany) in washing buffer (0.05 M Tris-HCL, pH 7.4, containing 150 mM NaCl and 1% Triton X-100), 30 min at 37 °C). Following this, the wells were incubated (30 min, 37 °C) with test samples serially twofold diluted (1:50–1:6.400) in washing buffer. Antibodies were detected using horseradish peroxidase (HRP)-conjugated rabbit anti-human IgG (DAKO, Glostrup, Denmark). The wells were washed x4 between each incubation step. O-phenylenediamine (OPD, Dako) served as substrate. Colour development was stopped (1M H_2_SO_4_, after 30 min) and read as OD at 492 nm (OD_492_). The positive control used was serum from a 10-year-old child who had had a *H. tarandi* larva removed from an eye [16] (collected in December after the summer of infestation). The negative control used was serum from a 50-year-old adult with no history of *H. tarandi* infestation. The controls were run in every plate. Each OD_492_-reading was expressed as an ELISA percentage relative to the 1/50-dilution reading for the positive (100%) and the negative (0%) control [19]. Identification of seropositives was by comparison with the corresponding negative-control dilution series in the same plate. Five additional sera from adults with no history of *H. tarandi* myiasis were assayed, and the dilution curves (1:50–1:6.400) for all were practically identical to the negative control curve. Each of the negative control dilutions gave an ELISA percentage within the range 5 to minus 5 and followed the negative control graph (Figure 1).

Symptom onset: October 2013. Serum samples assayed (ELISA) for antibodies (IgG) against Hypodermin C. First sample (October 2013) giving suspicion of myiasis, second sample (14 days later in November 2013) confirming seropositivity.

Seropositivity or seronegativity was identified by comparison with the dilution curve for the negative control. For test sera, if the 1/50-dilution ELISA percentage was in the 10–30% range, the serum was considered suspect, and seropositivity was confirmed or disproved by a new sample collected ≥ 2 weeks after the first. Seropositive samples gave ELISA percentages higher than the corresponding negative control dilution percentage, at dilutions of up to 1/1.600–1/3.200 or higher.

### Determination of larva and egg remnants

Larva identification was by morphology of cuticular spikes and mouthparts [10]. Egg remnants were identified by comparison with *H. tarandi* eggs.

## Results

**Figure 2:**
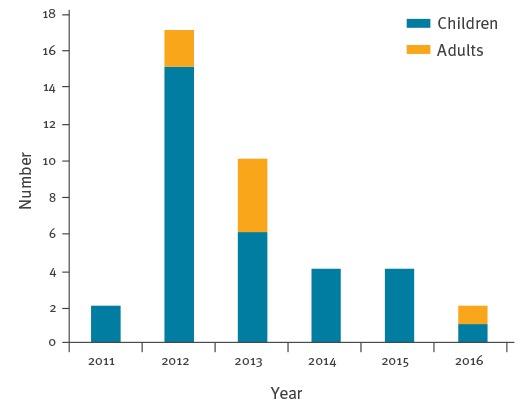
Myiasis cases caused by *Hypoderma tarandi*, by year, children and adults, Norway, 2011–2016 (n = 39)

The study included 39 persons from Norway seeking medical help for clinical symptoms compatible with *H. tarandi* myiasis from August 2011 to October 2016 (Figure 2). All had dermal swellings and all were seropositive.

The children ranged in age from 3 to 12 years, and the adults from 20 to 66 years. 2011 cases were diagnosed retrospectively in 2013.

Thirty-two cases were children (age range: 3–12 years, mean ± standard deviation (SD): 7.6 ± 2.4 years, 13/32 girls) and seven were adults (age range: 20–66 years; mean ± SD: 46.3 ± 16.8 years; 3/7 women). Thirty-two were resident in Finnmark. One of the adults, a traveller to Finnmark for hunting (August 2012, symptom onset September 2012), was seropositive by the first sample assayed (serum collected February 2013). Two children, with symptom onset in September and November 2011, respectively, were found to be seropositive by samples collected in February and January 2013, respectively. These three persons all sought medical help for their symptoms after receiving information via news media. One of the children had a dermal swelling on presentation (incised with no finding of larva) and was given one dose of ivermectin [15]. One child no longer had symptoms on presentation and received no treatment. Of the 39 cases, 17 were infested in 2012, 15 of the 17 in Finnmark. Aside from the myiasis symptoms, all cases presented with good general health.

**Figure 3:**
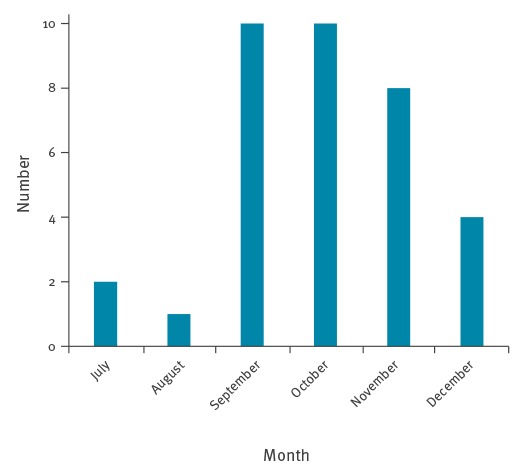
Month of symptom onset for myiasis cases caused by *Hypoderma tarandi*, Norway, 2011–2016 (n = 35)

Table 1 summarises the clinical characteristics. Figure 3 shows the time of symptom onset for 35 of the 39 cases.

**Table 1:** Clinical characteristics for myiasis cases caused by *Hypoderma tarandi*, Norway, 2011–2016 (n = 39)

Symptoms	Total
Dermal swellings^a^	39
**Number of dermal swellings**	
1	17
2–3	20
> 3	2
**Lymphadenopathy**	
Yes	25^b^
No	7
NA	7
**Eosinophilia**	
Yes	17
No	13
NA	9
**Identification of egg remnants**	1^c^
**Identification of larva**	1^d^
**Seropositive**	39
**Ivermectin treatment, number of doses**	
0	1^e^
1	21^f^
2	7
≥ 3	9
NA	1
**Ophthalmomyiasis**	0

NA: not available.

^a^ These included swelling in any of the following areas: forehead, temple, retroauricular area, occipital bone area, neck, jaw, cheek, nose, eyelid, scalp, temporal region of the skull. Periorbital oedema was seen in connection with swellings in 18 patients (14 unilateral, 4 bilateral), general oedema of face/forehead in four.

^b^ These included cervical, nuchal, occipital and retroauricular lymphadenopathy.

^c^ Egg remnants recovered from hair, identified as being from *Hypoderma tarandi*.

^d^ Live larva, recovered from a facial dermal swelling, identified as *H. tarandi*.

^e^ Myiasis symptom onset in 2011, no longer symptoms when diagnosed/confirmed seropositive in 2013.

^f^ Including a child (diagnosed 2016) with dermal swellings resolving spontaneously, who subsequently received one dose of ivermectin.

Symptom onset information not available for four cases.

In 35 of the 39 cases, seropositivity was confirmed by the serum collected at the first medical evaluation. In four cases, the serology result for the first serum sample (collected in August in one case, in October in three cases) was suspect, and seropositivity was confirmed by a new sample collected ≥ 2 weeks after the first. Another three cases had symptoms during autumn/winter, and serum collected at the first medical evaluation (in February, May and June, respectively) confirmed seropositivity.

Eight of the 39 cases were members of reindeer-husbandry families. Only one of the 39 (adult, seeking medical help in early July 2016) had noticed any contact with a bumble-bee-sized insect compatible with *H. tarandi* that could be associated with the infestation. This person observed a fly in their hair, and recognised it as *H. tarandi*, while ear-tagging reindeer calves in late June.

**Figure 4:**
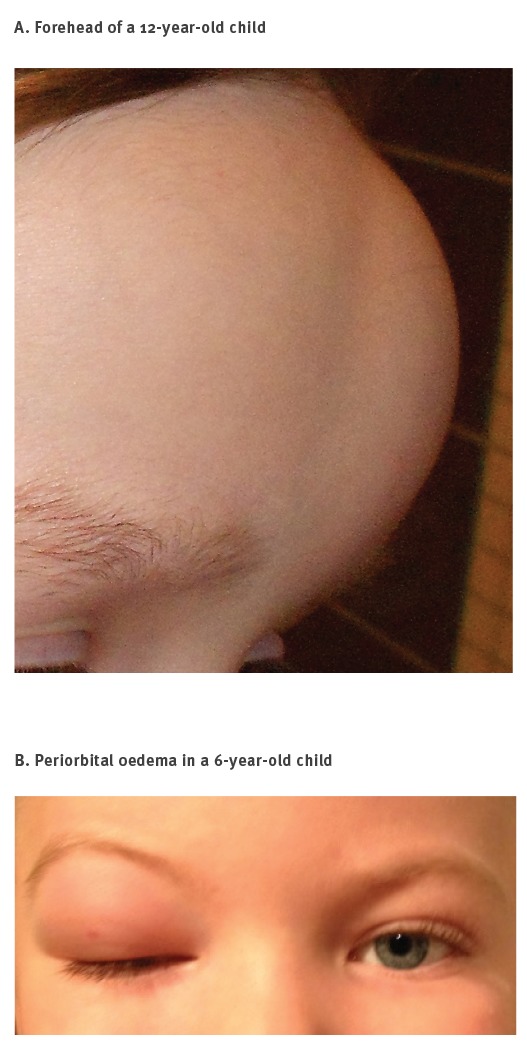
Dermal swellings in two cases infested with *Hypoderma tarandi*, Norway, October to November 2012

Dermal swellings (Figure 4a) were often migratory. Periorbital oedema (uni- or bilateral) (Figure 4b), in connection with dermal swellings, was commonly observed, and in a few cases was observed as a general oedema of the forehead.

4B: The oedema became bilateral one week later.

There was no apparent correlation between eosinophilia and severity of symptoms among the cases with eosinophilia (Table 1).

**Figure 5:**
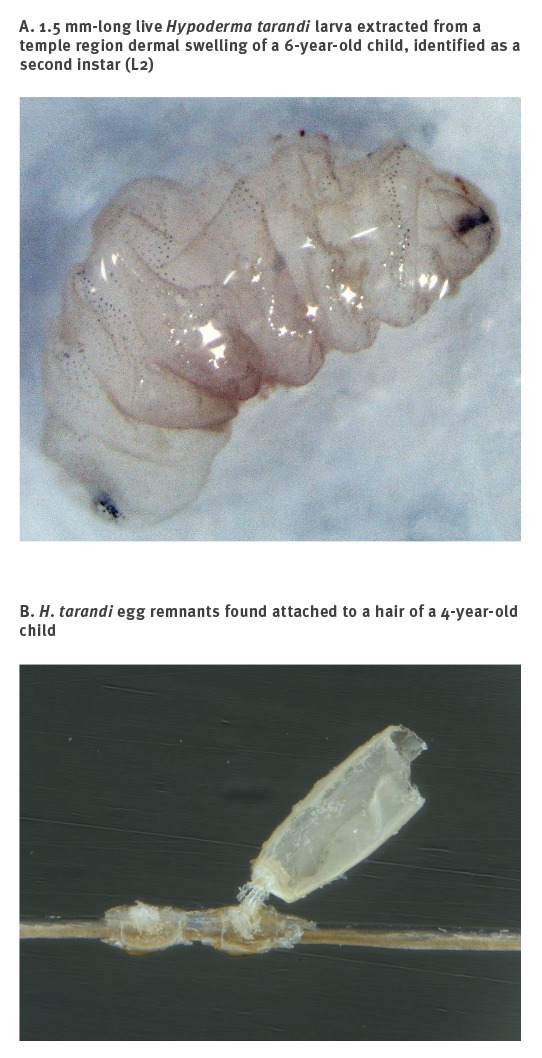
Larva and egg remnants in two cases infested with *Hypoderma tarandi*, Norway, September and November 2012

Three children had facial dermal swellings, each with a dark spot, suspected to be the larval breathing hole. For two of them, no larva or remnants thereof were found by incision of the swellings. From the third, a 1.5 mm-long live larva (Figure 5a) was extracted from a temple region swelling in mid-November.

The larva was identified as a second instar (L2, see Discussion) larva of *H. tarandi*. From another patient, egg remnants (Figure 5b) were found attached to scalp hairs in mid-September. The remnants were identified as being from *H. tarandi* eggs. The first 10 cases diagnosed in 2012 were referred to an ophthalmologist, who demonstrated no ocular complications (personal communication, Kristian Fossen, January 2013).

## Discussion

This report presents, to our knowledge, the largest case series of myiasis diagnosed in one geographic area of Europe. We assume that the information about *H. tarandi* myiasis that was broadcast through media and conference presentations from late 2012 to 2013 reached a large audience in the Nordic countries. In agreement with this assumption, the visitor from a southern Norwegian county to Finnmark for hunting in August 2012, sought medical help after receiving information via the media. Similarly, two children with symptom onset in September and November 2011, respectively, were diagnosed after their families had received information via the media, more than a year after symptom onset. Our serological assaying for *H. tarandi* myiasis was established in association with a case in Sweden in 2009 [20], and today there is still no other option for serological assaying for the condition in the Nordic countries. We therefore believe that we received blood samples from all suspected cases in 2011–2016, and that the 39 cases reported here make up the complete list of diagnosed cases in Norway in the period.

Most cases were residents of Finnmark, a county where the number of reindeer was 183,600 in 2012 [21], ca 2.5 times the number of humans. Ivermectin is regularly used in reindeer, targeting *H. tarandi* and other parasites, mostly to help the individual animal through the harsh arctic winter. Particularly in Finland, ivermectin has been used regularly since it came on the commercial market in the early 1980s. The long-term use of the drug does not seem to have reduced the *H. tarandi* population or the infestation problem in reindeer. In reindeer calves in Finland, the annual infestation level is up to 100% in the northernmost part of the reindeer-husbandry area [22]. Likewise, examination of 1,305 hides from slaughtered reindeer in Finnmark in 1984–1985 demonstrated *H. tarandi* larvae in 99.9% [23], and there is no indication of reduced infestation level in reindeer in Finnmark after that time. With such a high infestation level, it would be difficult to know whether the infestation level in reindeer was even higher in 2012 and 2013. It is a core assumption of parasite population dynamics that there is a positive relationship between parasite transmission rate and host population density [24]. Because Finnmark has a higher number of reindeer per inhabitant than any other region of Scandinavia and Finland, a higher frequency of human infestations could possibly be expected. There could, however, be other factors contributing to the higher number in Finnmark compared with other regions of Norway, such as differences related to weather and climate. Most cases were children, in common with reports of myiasis from other parts of the world [3,4], and we could speculate that differences such as immunity, skin thickness and behaviour between children and adults are possible affecting factors.

The cases presented with a uniform clinical picture involving enlarged occipital and retroauricular lymph nodes and dermal swellings located on the head and face, with symptom onset most often occurring between September and December. The main *H. tarandi* oviposition period is from July to August [25], and two cases with symptom onset in July clearly illustrate a risk of infestation as early as June. All 39 cases had dermal swellings. A large proportion of OMI in previously reported cases [6-17], along with the absence in most of these cases of reporting of dermal swellings, suggest that clinicians, including ophthalmologists in many cases, may have overlooked this important symptom. Consequently, patients’ delay in seeking medical help, and doctors’ delay in diagnosis, may have led to severe eye complications. The dermal swellings resolved spontaneously in two cases, indicating that the condition can be self-limiting. This was indirectly confirmed by primary care physicians in Finnmark, who told us in interviews that dermal swellings and lymphadenopathy had occasionally been observed over the years and were considered to be a condition of unknown origin, associated with autumn, that could heal spontaneously.

Ivermectin is the drug of choice in treatment of onchocerciasis (river blindness), strongyloidiasis, cutaneous larva migrans and other conditions caused by parasites [26]. The drug is generally well tolerated, as also demonstrated by our results. It has some protective effect on visual field loss and optic nerve disease in river blindness [27]. However, severe adverse effects are infrequently seen in river blindness treatment when substantial amounts of microfilariae are present, and serious post-treatment reactions, characterised by hypotension, pruritic rash, lymphadenopathy, arthralgia and malaise, was associated with treatment of river blindness especially in patients co-infected with the filarial nematode *Loa loa* [26,28,29]. There has been some uncertainty regarding the safety of ivermectin in the presence of ocular symptoms due to the possible risk of local inflammatory reaction [13]. Ivermectin given in a case of OMI resulted in visual improvement, with no serious side-effects noted [30].

The live larva extracted from a dermal swelling of a child was identified as a second instar (L2) larva. The larva of *H. tarandi* has three developmental stages (L1-L3). L2 encysts in a nodule under the skin, with a breathing hole to the outside, where it develops to L3, the stage where it leaves the host through the breathing hole [5]. The dark spot on top of the dermal swelling, suspected to be the larva breathing hole, indicated stage L2 [31], as did the size and morphology of the larva. Development to *H. tarandi* L2 in a human has not, to our knowledge, been reported before. *H. tarandi* eggs in the hair, and egg remnants as demonstrated in one case here, are easily distinguished from head lice eggs [20].

Of the 39 cases, 31 were not members of reindeer husbandry families or in other potentially close contact with reindeer. For the Sami people, an indigenous population in Norway, Sweden, Finland and the Kola peninsula of Russia, reindeer husbandry is an important part of their way of life. There is no indication that Sami have any raised awareness of the risk of human infestation by *H. tarandi* (data not shown). Since *H. tarandi* oviposits on hair, the prevention measure we suggest to avoid infestation is to wear a hat or similar, and indeed, Sami people have a long tradition of covering their hair when outdoors.

Until recently, human myiasis caused by *H. tarandi* was largely unknown to physicians and general public in the Nordic countries. Knowledge of a recently reported case in Sweden [20] was important for the diagnosis of the first cases in Finnmark in 2012. These cases drew the immediate attention of mass media in Norway, Sweden and Finland. Although Sweden and Finland have high numbers of reindeer, we are not aware of any reported human case in Finland, or any new case in Sweden since 2011. A case in Greenland was reported in early 2016 [17]. The diagnosis of the many cases in 2012 and 2013 could partly be due to increased awareness of the condition after the recent reporting of cases from Sweden [15,20]. It is not likely that the many diagnoses were due to the publicity in the media and the letter to physicians about *H. tarandi* myiasis, since the publicity and the letter came after the first three cases were diagnosed in early November 2012. Furthermore, since there were so many cases diagnosed over a short time (infestation season 2012; 12/17 diagnosed in November–December 2012, 5/17 in January–June 2013, data not shown), we consider the cases after the infestation season 2012 to be an outbreak, possibly caused by weather and climate conditions or other factors. Such factors could also possibly contribute to explaining the apparent difference in prevalence of myiasis caused by *H. tarandi* between countries and regions, such as between Finland and northern Norway. This issue, and the apparent difference in prevalence between children and adults, is beyond the scope of this study and awaits further evaluation.

In western and southern European countries and in North America, most cases of myiasis are seen in travellers returning from sub-Saharan Africa, Central and South America [3,4]. The aetiology of myiasis in patients with no history of recent travel to a tropical or subtropical country can be a diagnostic problem [3]. In addition, migratory myiasis is considered more difficult to diagnose compared with the furuncular myiasis because of the absence of a breathing hole [31]. Healthcare professionals, in regions where humans have a likelihood of being infested with *H. tarandi*, and those meeting travellers returning from such regions, should be aware of the condition, and they are advised to obtain a detailed travel history when assessing patients with migratory dermal swellings.

## Conclusion

The diagnosis of myiasis in patients with no history of recent travel to a tropical or subtropical country can be a diagnostic problem. Myiasis caused by *H. tarandi* is more common than previously thought, at least in Norway, but the prevention measure is simply to cover the hair with a cap or similar. The condition presents a uniform clinical picture that should be recognised by physicians in areas populated by reindeer and those meeting travellers returning from such regions. Ivermectin should be given on clinical suspicion. If symptoms persist after the first dose, the treatment should be repeated. Serological confirmation is recommended when possible. Only patients with ocular symptoms should be referred to an ophthalmologist.
